# A human rights approach: The right to education in the time of COVID‐19: Commentary on COVID‐19 Special Section

**DOI:** 10.1111/cdev.13654

**Published:** 2021-08-24

**Authors:** Sandra Fredman

**Affiliations:** ^1^ University of Oxford Oxford UK

One of the most serious consequences of the COVID‐19 pandemic has been the disruption of children's education worldwide with the closure of schools for public health reasons. Projections from UNESCO Institute for Statistics show that nearly 100 million children across eight age cohorts would move below the minimum proficiency threshold in reading in 2020 due to the pandemic (UNESCO Institute for Statistics, 2021). Both current studies and experience of school closures due to previous similar crises, such as the Ebola epidemic, show that COVID‐19 closures risk exacerbating vulnerabilities for those who are already disadvantaged (Azevedo et al., 2021). This includes lack of access to the vital nutrition provided by school nutrition programs (Borkowski et al., 2021); exposure to violence at home; early marriages and pregnancies for girl children (De Paz et al., 2020); lack of social interaction (Larsen et al., 2021); and deepening inequalities for those without access to the Internet (United Nations Children’s Fund & International Telecommunication Union, 2020).

While the serious consequences of these disruptions are well recorded, less attention has been paid to the human rights breaches entailed. Governments throughout the world have ratified international human rights treaties which enshrine the right to education, together with the rights to equality and non‐discrimination, privacy, life, food, personal security, and housing. These are not just political promises as in the Sustainable Development Goals, but create legally binding obligations on States. This paper examines and elaborates the legally binding human rights obligations in relation to the right to education and non‐discrimination, which should provide the basis for governments to determine their priorities and allocate their already strained resources for emerging from the pandemic. A human rights‐based approach is essential to ensure that measures taken to mitigate the effects of the pandemic both now and in the future do not exacerbate inequalities in access to the right to education, and fully comply with States’ legally binding commitments in international human rights law.

The right to education is found in three international human rights treaties, the Convention on the Rights of the Child ("CRC," 1989), the International Covenant on Economic, Social and Cultural Rights ("ICESCR," 1966), and the Convention on the Rights of Persons with Disabilities ("CRPD," 2006). The CRC has been ratified by 196 out of 197 States, excluding only the United States; ICESCR has 171 ratifications; and CRPD has 182. Ratifying States are obliged to ensure that their domestic law complies with the treaty's provisions: States which fail to comply will be in breach of international law. While the United States has signed all three Covenants, it has not ratified them. Signing without ratifying a treaty does not establish consent to be bound. However, it creates an obligation on the signatory State to refrain from acts which would defeat the object and purposes of the treaty ("Vienna Convention," 1969), Art, 18. The United States cannot, therefore ignore its obligations under the three Conventions.

The COVID‐19 pandemic has not extinguished States’ binding obligations under the Covenants. Three key aspects should be highlighted. First, the obligation to fulfill the right to education comes with a duty to do so without discrimination on a range of grounds, including race, gender, disability, language, national or social origin, birth, or “other status” (CRC, 1989, Art. 2; CRPD, 2006, Art. 4; ICESCR, 1966, Art. 2), such as economic and social situation (Committee on economic social and cultural rights, 2009). This duty is particularly salient during the current pandemic, when deep pre‐existing inequalities have been magnified and intensified. While States have instituted mitigating measures, such as online teaching, these have also exacerbated inequalities in access to the key infrastructure for such learning, including access to Internet, computers, housing, and parental support. UNESCO has reported that half of all learners who could not go to school due to the pandemic had no access to a household computer and as many as 43% have no Internet at home, a figure which reaches as much as 82% for sub‐Saharan Africa (UNESCO, 2020). Girls’ are often allowed less access to technologies than boys, and children with visual and hearing impairments are frequently excluded. In addition, parents, teachers, and learners may not have the skills to use these technologies (Education International, 2020).

In emerging from the pandemic, the non‐discrimination duty will not be fulfilled merely by mitigating the extra burden falling on disadvantaged groups due to the pandemic itself. States are also required to redress the structural deficits in education which made it inevitable that a pandemic would exacerbate inequality. The UN Special Rapporteur on the Right to Education (UNSR) in her report on the impact of COVID on the right to education, emphasizes that the numerous innovative measures adopted by governments could not compensate for “past failures to build strong and resilient education systems and to fight entrenched inequalities” (Special Rapporteur on the Right to Education, 2020). A human rights compliant framework requires States to ensure that they understand and address the factors contributing to the increased discrimination in the enjoyment of the right to education during the crisis, including the consequences of poorly funding educational institutions.

The second key element concerns States’ duty to devote the “maximum of their available resources, individually or through international assistance and co‐operation” to the fulfillment of the right to education (CRC, 1989, Art 4; CRPD, 2006, Art 4(2); ICESCR, 1966 Art 2(1)). Conversely, if any deliberately retrogressive measures are taken, the State Party must prove that this is only after the most careful consideration of all alternatives and in the context of the full use of the State Party's maximum available resources (Committee on economic social and cultural rights, 1999). Until recently, treaty bodies responsible for monitoring compliance with the Conventions (treaty bodies) tended to assess available resources only by reference to States’ chosen budgets and international assistance (Balakrishnan et al., 2011). In the last decade, however, treaty bodies have indicated that States also have an obligation to mobilize resources, including by investment in employment, education, and health. Importantly, investment in accessible and quality education is regarded as a measure that both protects individual rights and increases a state's assets and therefore the resources available to support human rights in the long‐term (Independent Expert on the effects of foreign debt and other related international financial obligations of States on the full enjoyment of human rights, 2016). Nor is this an obligation States are required to carry out single‐handedly. Wealthier States have a duty of co‐operation and assistance, and treaty bodies have endorsed the UN recommended target of the expenditure 0.7% GNI on Overseas Development Aid (Committee on economic social & cultural rights, 2017). This is, however, only a weakly developed obligation in international human rights law. Given that governments, such as the United Kingdom, are now using the pandemic as a pretext for swingeing cuts to Overseas Development Aid, there is an urgent need for this obligation to be clarified and strengthened in treaty bodies’ compliance efforts.

As well as requiring a wider mobilization of resources to fulfill human rights, States are obliged to use their resources efficiently and to distribute them fairly. There has been a strong presumption in recent decades that efficiency is enhanced by the privatization of education, and especially through low‐fee and for‐profit private schools. The World Bank, the largest external funder of education in poor countries, has a policy of actively advising countries to expand private education provision through public–private partnerships (PPP) and for‐profit schools, including making loans and other funding conditional on expanding funding for PPP. An Oxfam study found that 22% of World Bank funding to governments for primary and secondary education between 2013 and 2018 included direct support for private provision (Bous, 2019). However, the evidence shows that low‐fee and commercial private schools do not provide a path to quality education for all, as required by the right to education. School fees, even those considered low, restrict girls’ access to schooling, running counter to the huge increases in girls’ entry to school with the widespread elimination of user fees at public schools since 2000 (United Nations, 2015). Research in Pakistan and Uganda found that World‐Bank supported PPP schools were not affordable to the poorest children, especially girls and pupils with disabilities, and that the quality of education was poor (Bous, 2019). Moreover, low fee private schools cut costs by paying very low salaries to teachers and using poorly qualified teachers, the majority of whom are women. Particularly worryingly, these initiatives allow States to abdicate responsibility for public education and deplete State funding. While the ICESCR requires States to respect the liberty of parents to choose private schools, States must ensure that such schools conform to minimum educational standards (ICESCR, 1966, Art 13(3)), and are not required to fund them (Fredman, 2021). This liberty to establish private schools should not interfere in any way with the duty on States to provide free compulsory, non‐discriminatory primary education.

The UNSR has expressed particular concern at the situation in private schools during and after COVID. Since the economic model of such institutions is heavily reliant on the payment of fees, many have imposed pay cuts or compulsory leave without pay on their staff, even though they continue to work from home (Special Rapporteur on the Right to Education, 2020). Particularly worrying are reports suggesting a collapse of low‐fee private schools in Pakistan and other countries, resulting in increased pressure on the public system to enroll such children when they re‐open. As the UNSR concludes, this is a clear demonstration of the limitations of education models based on privatization and commercialization and re‐emphasizes States’ central human rights obligation to “prioritize the funding and provision of free, quality, public education” (Special Rapporteur on the Right to Education, 2020). Also of concern is the need for proper control and regulation of the many private actors who have entered the educational field through digital technologies. Such private actors should not be permitted to capture limited public resources required to be spent on education, and other options such as public online learning platforms should be enhanced (Special Rapporteur on the Right to Education, 2020).

The third key element concerns the nature of States’ duties. Although the full realization of the right to education might not be possible immediately, States have a “specific and continuing obligation to move as expeditiously as possible towards the full realization” of the right (Committee on economic social and cultural rights, 1999). Importantly, non‐discrimination is an immediate duty, which cannot be delayed. The closure of schools during the COVID‐19 pandemic magnified States’ obligations to provide access to schools. However, States’ obligations extend beyond access. States are also required to ensure the provision of functioning educational institutions with sanitation, safe drinking water, trained teachers, IT, and computer facilities. Schools must also be accessible, physically, and economically; acceptable in form and substance; and adaptable to changing needs (Committee on economic social and cultural rights, 1999). Ultimately, States must ensure education is directed to the overriding aims expressed in the Covenants, which include both the development of the child's personality and abilities to their fullest potential, and their preparation to participate effectively in society “in the spirit of understanding, peace, tolerance, equality of sexes, and friendship among all peoples …”. (CRC, 1989, Art. 13; CRPD, 2006, Art 24; ICESCR, 1966, Art. 29(1)(a) and (d)). Online teaching only very partially achieves these objectives, and then only for a minority of learners. The use of digital tools, while bringing important benefits, also needs to be managed in a human‐rights compliant way. In particular, children's right to privacy requires States to protect their education data, especially in relation to the collection, retention, or sharing of such data (Special Rapporteur on the Right to Education, 2020), for commercial, immigration, or security purposes.

For our youngest generations, the loss of education during the pandemic has been a heavy burden, and its consequences will linger in years to come. Moreover, the pandemic exposed the deep underlying inequalities and weaknesses in the system, as well as triggering further potential human rights breaches. The promise of “building back better” once we emerge from this pandemic should have the right to education as a central pillar. This is not just a good policy choice for both individuals and societies. It is a binding obligation in international human rights law.
